# A Case Report of Exercise-Induced Pulmonary Oedema After an Ironman

**DOI:** 10.7759/cureus.97343

**Published:** 2025-11-20

**Authors:** Chloe Thomas, Meet R Vaghela, Syed Bokhari

**Affiliations:** 1 Department of Medicine, Withybush General Hospital, Haverfordwest, GBR

**Keywords:** athletes, endurance sport, exercise-induced pulmonary oedema, hypoxia, non-cardiogenic pulmonary oedema

## Abstract

Exercise-induced pulmonary oedema (EIPO) is a rare and poorly understood condition, most commonly reported in patients participating in swimming-based endurance events. We report a 47-year-old male who developed acute dyspnoea and a cough during the cycling segment of an Ironman triathlon post swimming stage. The patient completed the race with his symptoms, and post-race assessment revealed hypoxia, prompting transfer to the local emergency department. Chest imaging showed acute pulmonary oedema. The patient was treated with furosemide and made a full recovery. EIPO is relatively uncommon in sporting events and typically presents with dyspnoea and hypoxia. It is most frequently observed in cold-water, submersive sports, although cases during land-based endurance activities have been reported. The underlying pathophysiology remains poorly understood. With the growing popularity of endurance sports, it is essential for clinicians to recognise EIPO promptly to ensure timely management.

## Introduction

Professional-level endurance sports impose intense physical and psychological stress on the body within a short time frame and can acutely precipitate metabolic derangements. One such rarely described phenomenon is exercise-induced pulmonary oedema (EIPO), a condition hypothesised to result from transient increases in pulmonary capillary pressure, leading to fluid leakage from capillaries into the alveolar interstitium and impairing efficient gas exchange. Most cases have been reported in swimmers, cyclists and runners [1]. Our case report presents a patient with EIPO after the completion of an Ironman race. An Ironman consists of a 2.4-mile swim, a 112-mile bike ride, followed by a 26.2-mile run. His condition was recognised quickly and appropriately acted upon by medical professionals, with conservative management. This case illustrates the importance of early recognition of EIPO in endurance athletes.

## Case presentation

Our case describes a previously healthy 47-year-old man who presented to the emergency department (ED) of a rural district general hospital in Wales, approximately six to seven hours after the acute onset of dyspnoea and a new, persistent cough during his Ironman race. In the first stage of his race, whilst swimming, the patient reported swallowing a significant quantity of water owing to the turbulent water and weather conditions. Following on from this to the cycling stage, the patient first noticed dyspnoea and a new persistent cough, but continued to complete the race. 

Upon assessment post-race, the patient was still suffering from worsening dyspnoea. The event medics found widespread crepitations in his chest up to the midzones bilaterally, and the man to be saturating at 82% on room air. Following oxygen therapy, his saturations improved but persistently dropped <90% when taken off oxygen; owing to these alarming examination findings and new oxygen requirement, he was transferred to Withybush General Hospital for further assessment and management.

Upon presentation to the emergency department, the emergency medicine team confirmed the previously noted chest findings, observed that his heart was in sinus rhythm with no additional murmurs, found no pedal oedema and his abdomen to be unremarkable. His oxygen saturations were 82% on room air and was given 15 L of supplemental 100% oxygen via a non-rebreather mask. 

His observations on the initial assessment were recorded: respiratory rate: 19, oxygen saturation: 100% on 15 L non-rebreather mask, heart rate: 71, blood pressure: 119/81, and temperature: 36.6°C. His National Early Warning score was 2 due to his oxygen requirement. His ECG showed a right bundle branch block, but he was in sinus rhythm and no ischaemic changes. His blood results on admission are shown in Table 1. They demonstrate a raised white cell count, high-sensitivity troponin T, d-dimer and B-type natriuretic peptide (BNP). The arterial blood gas (ABG) showed hypoxaemia.

**Table 1:** Laboratory investigations during admission, showing mild leukocytosis and elevation of inflammatory and cardiac biomarkers with normal renal function. eGFR: estimated glomerular filtration rate; g/L: grams per litre; pg: picograms; fL: femtolitres; U/L: units per litre; mg/L: milligrams per litre; ng/L: nanogram per litre; mmol/L: millimoles per litre; μmol/L: micromoles per litre; ×10^9^/L: billions per litre; ×10^12^/L: trillions per litre; mL/min/1.73 m^2^: millilitres per minute per 1.73 square metres of body surface area; kPa: kilopascal. NT-proBNP: N-terminal prohormone of brain natriuretic peptide.

Parameter	Result	Reference range	Unit
White blood cell count	13.1	4.0-11.0	×10^9^/L
Neutrophil count	11.4	1.7-7.5	×10^9^/L
Lymphocyte count	1.3	1.0-4.5	×10^9^/L
Monocyte count	0.4	0.2-0.8	×10^9^/L
Eosinophil count	0.0	0.0-0.4	×10^9^/L
Basophil count	0.0	0.0-0.1	×10^9^/L
Haemoglobin	138	130-180	g/L
Red blood cell count	4.70	4.50-6.00	×10^12^/L
Haematocrit	0.40	0.40-0.52	L/L
Mean cell volume	86	80-100	fL
Mean cell haemoglobin	29.3	27.0-33.0	pg
Red cell distribution width	12.9	11.0-14.8	%
Platelet count	163	150-400	×10^9^/L
Prothrombin time	12.3	9.0-13.0	Sec
Activated partial thromboplastin time	28.4	27.0-36.4	Sec
Claus fibrinogen level	3.2	2.0-4.0	g/L
D-dimer	1294	H<500	
High sensitivity troponin T level	24	H<14	ng/L
NT-proBNP	848		ng/L
Bilirubin	20	<21	
Total protein	62	60-80	g/L
Albumin	41	35-50	g/L
Globulin	21		g/L
Alkaline phosphatase	70	30-130	U/L
Alanine transaminase	132	H<41	U/L
C-reactive protein	46	H<5	mg/L
Urea	6.8	2.5-7.8	
Creatinine	75	58-110	
Estimated GFR	>90	>90	mL/min/1.73 m^2^
Sodium	140	133-146	mmol/L
Potassium	4.5	3.5-5.3	mmol/L
pH	7.46	7.35-7.45	
PaO_2_	7.77	11-13	kPa
PaCO_2_	5.13	4.7-6.0	kPa

**Figure 1:**
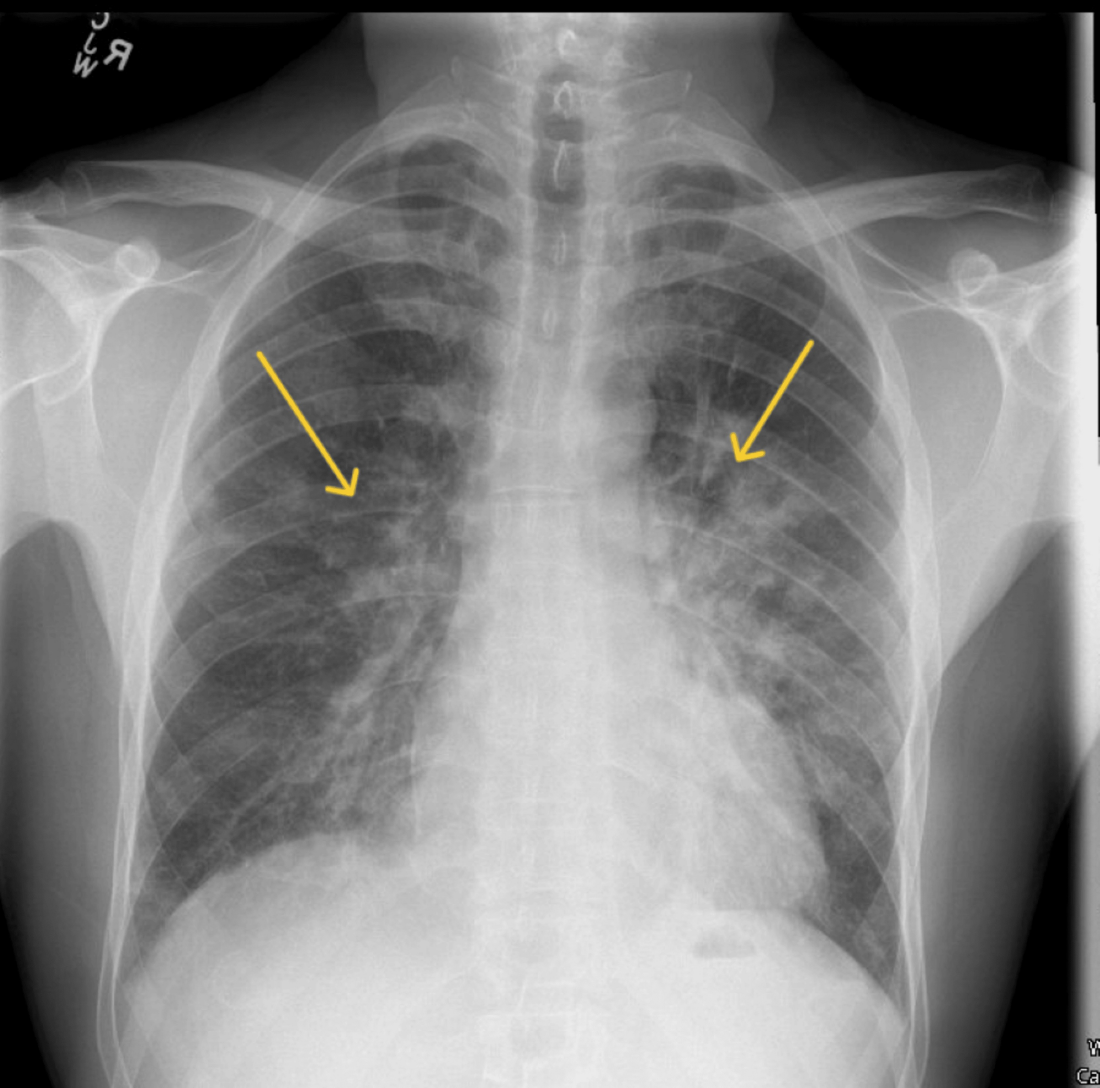
Chest X-ray demonstrating bilateral patchy alveolar infiltrates, most pronounced in the perihilar and lower lung zones (arrows), consistent with pulmonary oedema.

The chest X-ray showed bilateral patchy perihilar infiltrates consistent in appearance with pulmonary oedema (Figure 1).

Echocardiography showed a non-dilated left ventricle with normal systolic function, a Simpson's bi-plane ejection fraction of 58%, and a non-dilated right ventricle with preserved systolic function. A dilated mid-ascending aorta at 3.8 cm with trivial aortic regurgitation.

Collating the examination findings with the laboratory results, the patient was treated for acute pulmonary oedema in the emergency department. He received an immediate dose of 40 mg intravenous furosemide and a supplemental 960 mg immediate dose of co-trimoxazole to cover for chemical pneumonitis secondary to water inhalation or ingestion during the swimming stage of his Ironman event.

Due to blood results demonstrating a high BNP and troponin, reflecting cardiac damage, in combination with bilateral perihilar infiltrates seen on chest x-ray, and symptoms resolving after furosemide administration, differential diagnoses such as aspiration pneumonia and acute respiratory distress syndrome were excluded. The absence of ischaemic changes on ECG ruled out other cardiogenic causes of the presentation. 

The following day, the patient was reviewed by the cardiology team. His dyspnoea was resolving, and he was successfully weaned off supplemental oxygen. He was subsequently discharged after a three-day hospital admission, with an outpatient cardiac MRI arranged, and cardiology follow-up scheduled. He was also advised to refrain from participating in future endurance exercises until his review.

## Discussion

Previous case reports have described the development of EIPO in athletes [1]. This condition is most documented with submersive sports, such as scuba diving and long-distance swimming, particularly in cold-water environments, where increased pulmonary vascular pressures and peripheral vasoconstriction place additional stress on the lungs [2]. Its occurrence during land-based activities, such as cycling and running, is more controversial; however, a small number of cases have been documented involving individuals participating in ultramarathons and endurance cycling events. A case report in the literature closely resembling ours described a previously healthy individual who developed symptoms of EIPO during the swimming segment of a triathlon and was subsequently unable to complete the event due to the severity of his symptoms [1].

The exact pathogenesis of EIPO remains poorly understood. However, it is hypothesised that the rapid accumulation of oedema within the alveolar interstitium and alveolar gas-exchange surface results from elevated cardiac output and high capillary perfusion pressures. This is driven by a combination of peripheral vaso- and venoconstriction occurring during exercise, leading to greater venous return, causing central blood pooling and greater cardiac output [3]. This is further augmented by the potent vasoactive effects of systemic catecholamine release, further increasing cardiac output by increasing inotropy and chronotropy. The combination of high preload, elevated cardiac output, peripheral vasoconstriction, and increased venous return generates elevated pulmonary circulatory pressures, promoting a transudative process at the alveolar capillaries that allows fluid to leak into the interstitium and alveolar gas-exchange surface. This hyperdynamic circulatory state, particularly during cold-water exercise, leads to a clinical ventilation-perfusion (V/Q) mismatch, manifesting with dyspnoea and cough as seen in our case report [4,5].

EIPO is often associated with rapid recovery and minimal long-term sequelae. Most reported cases involve an inpatient stay of approximately one day, with prompt resolution of symptoms following administration of furosemide. A previous study reported a recurrence rate of 23%, highlighting the importance of counselling patients regarding the risk of recurrence and advising them to seek immediate medical attention should similar symptoms arise during future sporting activities [6]. 

## Conclusions

This case report underscores the importance of recognising the clinical features of EIPO and raising awareness of the condition among emergency medicine, cardiology, and event doctors. It highlights the need to manage symptoms, such as dyspnoea and new-onset cough, appropriately in endurance medicine, rather than reflexively treating patients for chemical pneumonitis or acute respiratory distress syndrome when clinical examination, biochemical and radiological findings all suggest pulmonary oedema. 

EIPO can typically be managed quickly and safely with furosemide, with most patients achieving complete recovery due to the absence of underlying structural pathology. A recurrence has been reported, and patients should be counselled regarding this risk as part of discussions about prognosis and outcomes. 
